# Exploring Genital Lichen Sclerosus: Navigating from Pathophysiology to Precise Diagnostic Approaches

**DOI:** 10.3390/biomedicines13092252

**Published:** 2025-09-12

**Authors:** Maja Sever, Katarina Trčko, Tanja Zidarič, Tina Maver

**Affiliations:** 1 Faculty of Medicine, University of Maribor, Taborska ulica 8, 2000 Maribor, Slovenia; maja.sever98@gmail.com; 2 Department of Dermatology and Venereal Diseases, University Medical Centre Maribor, Ljubljanska ulica 5, 2000 Maribor, Slovenia; katarina.trcko@um.si; 3 Institute of Biomedical Sciences, Faculty of Medicine, University of Maribor, Taborska ulica 8, 2000 Maribor, Slovenia; 4 Department of Pharmacology, Faculty of Medicine, University of Maribor, Taborska ulica 8, 2000 Maribor, Slovenia; tina.maver@um.si

**Keywords:** lichen sclerosus, autoimmune dysregulation, sclerotic tissue, oxidative stress, genital disease

## Abstract

Lichen sclerosus (LS) is a chronic, relapsing skin disease that predominantly affects the perineal and genital regions, although extragenital manifestations can occur. Despite its significant impact on patients’ quality of life, particularly affecting sexual and urinary function, LS remains underdiagnosed. Multiple factors, including genetic predisposition, hormonal changes, immunological abnormalities, trauma, and urine irritation, contribute to its development and persistence. This review aims to clarify the complex pathophysiology of LS by exploring three main mechanisms: autoimmune dysregulation, sclerotic tissue formation, and oxidative stress. Autoimmune dysregulation involves T-cell infiltration and the roles of miR-155 and extracellular matrix protein 1 dysfunction, leading to chronic inflammation. miR-155 contributes to sclerotic tissue formation alongside galectin-7, promoting fibroblast proliferation and collagen synthesis. Oxidative stress results in tissue damage, autoimmunity, chronic inflammation, and an increased risk of carcinogenesis. Understanding these mechanisms is crucial for developing targeted therapies and improving LS management. Further research is needed to unravel the genetic basis, immune responses, and interactions between key mediators, ultimately advancing innovative therapeutic strategies and precision medicine in LS.

## 1. Introduction

Lichen sclerosus (LS) is a chronic and idiopathic skin disorder of unknown aetiology, marked by a progressive, relapsing nature. In rare instances, cases of remission have also been reported. While LS can affect any skin tissue, its primary manifestation occurs in the perineal and genital regions, where it is observed in 85% to 98% of cases. Extragenital involvement, though less common, occurs in approximately 15% to 20% of cases and may affect areas such as the face, neck, shoulders, trunk, and back. The initial presentation of LS typically includes asymptomatic, clustered white patches that gradually evolve into plaques, causing the affected skin or mucosa to resemble parchment in texture. Purpura or haemorrhagic spots often accompany these characteristic lesions. The prevalence of extragenital LS is likely underestimated due to its frequently asymptomatic nature, despite its occurrence generally aligning with that of genital LS [1,2,3,4].

Differentiating LS from other skin conditions is critical, as its clinical features can overlap with diseases such as lichen planus (LP), vitiligo, eczema, psoriasis, autoimmune blistering diseases, and candidiasis. One notable distinction between LS and LP is that LS rarely affects mucous membranes, including the oral and vaginal tissues, which are more commonly involved in LP [5,6,7].

In its early stages, LS appears as ivory-whitish atrophic patches or plaques with a waxy texture, often giving the skin or mucosa a cigarette paper-like appearance. Purpura, erosions, hyperkeratosis, bullae, and ulcerations can also be seen. As the disease progresses, LS can lead to scleroatrophy, adhesions, and scarring, which may cause significant disfigurement. This may result in progressive labial fusion, tearing of the tissue, clitoris burying, and intraorbital stenosis in female patients. Male patients typically have phimosis, foreskin adhesions to the glans, and meatal stenosis as sequelae. This can result in substantial impairment of urinary and sexual functions, greatly diminishing the quality of life for affected individuals [8,9]. Many patients experience itching, pain, and discomfort during intercourse, with 79% of women with LS reporting such symptoms [2]. In addition to sexual dysfunction, there is a high prevalence of anxiety among LS patients [10,11,12]. Studies show that women with LS engage less frequently in sexual activities, including vaginal intercourse, oral sex, and masturbation, compared to control groups [2]. Moreover, untreated LS carries a risk of malignant transformation [13,14,15], emphasising the importance of early diagnosis, appropriate treatment, and vigilant monitoring of disease progression [16,17,18].

Despite the significance of LS, treatment options remain limited, largely due to an incomplete understanding of the genetic, hormonal, and immunological factors that contribute to the disease. However, advances in molecular techniques, particularly in targeting specific disease-related molecules, offer hope for more effective treatments to halt disease progression and reduce the risk of malignancy. A deeper understanding of LS’s etiopathogenesis is essential for evaluating current therapies and developing innovative treatment strategies [6,18,19].

Conventional treatment of vulgaris LS relies primarily on highly potent topical corticosteroids, with clobetasol propionate 0.05% being the most commonly recommended agent. Its therapeutic efficacy is based on its dual anti-inflammatory and anti-fibrotic properties, which are achieved by inhibiting Th1-mediated cytokine activity and suppressing dermal collagen deposition [20,21,22,23]. In a double-blind, vehicle-controlled study, clobetasol showed complete clinical remission in all evaluable cases. Histopathological analysis revealed a significant reduction in hyperkeratosis, basal keratinocyte degeneration, and lymphocytic infiltration, with no evidence of steroid-induced skin atrophy or secondary infections [24]. Several randomised trials have further confirmed the superiority of clobetasol over other potent corticosteroids, such as mometasone furoate, and underlined the importance of structured dosing regimens to optimise efficacy and safety [20].

In addition, a longitudinal study of 507 women emphasised the clinical benefits of strict adherence to a staged clobetasol regimen (daily for 4–6 weeks, then alternating for 4–8 weeks, followed by twice-weekly maintenance treatment). Patients who adhered to this protocol showed significantly higher rates of symptom control (93.3% vs. 58.0%), no progression to vulvar intraepithelial neoplasia or carcinoma (0% vs. 4.7%), less scarring (3.4% vs. 40.0%), and minimal, reversible skin atrophy (1.1%). These results emphasise the crucial role of sustained suppression of chronic inflammation and skin remodelling for sustained symptom relief and attenuation of long-term malignant transformation in LS [21,22].

Despite the increasing research interest in LS, a consolidated overview of its clinical and pathological aspects remains essential. Based on clinical relevance and methodological quality, which includes systematic reviews, scoping reviews, clinical updates, and original studies, this narrative review summarises the results of a broad selection of peer-reviewed publications from 1999 to 2025, including high-impact journals in the fields of dermatology, gynaecology, and general medicine. As this is a narrative review, formal reporting frameworks such as PRISMA were not applied, allowing flexibility in integrating different evidence to provide a comprehensive clinical perspective on LS.

## 2. Epidemiology

The prevalence of LS is often underestimated. Studies suggest that LS affects between 1 in 300 and 1 in 1000 individuals in the general population based on dermatology visits [23]. Underestimation and misdiagnosis, particularly in the early, often asymptomatic stages, contribute to the challenge of accurately identifying the disease. Many patients, especially those with genital involvement, are hesitant to seek medical attention, which further exacerbates the under-recognition and undertreatment of LS in clinical practice [18,19].

LS can affect individuals of all ages and both sexes, although it is more commonly observed in adults than in children [24,25]. Notably, the disease may manifest in childhood but remain asymptomatic until adulthood [18]. In prepubertal women, vulvar LS typically manifests as itching, perineal discomfort, dysuria, and constipation, often due to fissures and oedema. Characteristic clinical findings include a “figure-8” pattern of ivory-white, atrophic papules affecting the labia minora, clitoral hood, and perianal region. Other features may include purpura, telangiectasia, and labial adhesions. Although symptoms may be inconspicuous or absent in the early stages, untreated paediatric LS often persists and recurs over time, which can lead to progressive fibrosis and anatomical distortion in adulthood [26,27]. While both males and females can develop LS, the condition disproportionately affects women, with a female-to-male ratio ranging from 3:1 to 10:1 [28]. In men, LS typically presents as lesions on the glans penis and prepuce, a condition also known as balanitis xerotica obliterans (BXO). In women, the lesions predominantly occur on the vulvar, perineal, and perianal regions [13].

**Figure 1:**
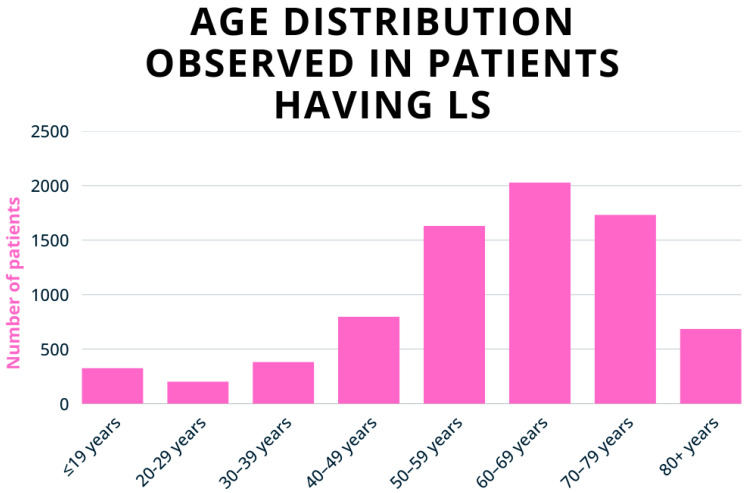
Age-specific incidence of vulvar LS per 100,000 women, according to Finnish register data (1969–2012). The incidence peaks at 7 per 100,000 in girls aged 5–9 and increases to 24–53 per 100,000 in postmenopausal women. Adapted with permission from Ref. [29], 2020, Wiley. https://doi.org/10.1111/1471-0528.16175 (accessed on 8 August 2025).

The age-specific incidence of LS shows a bimodal distribution, with a first peak occurring in girls aged 5–9 years and a second peak in postmenopausal women (Figure 1) [29]. This pattern is thought to reflect hormonal influences, particularly fluctuations in oestrogen levels. Hypoestrogenism can affect the integrity of vulvar skin by reducing hydration and downregulating the synthesis of type I and III collagen and glycosaminoglycans. These changes contribute to the chronic inflammation and sclerosis characteristic of LS lesions [30,31]. Recent studies, however, suggest that LS may be more prevalent among fertile women than previously thought, challenging earlier assumptions about its age distribution. The mean age at diagnosis is estimated to be around 32 years, with symptoms typically emerging around 27 [3,19,28].

## 3. Pathophysiology of LS

### 3.1. Disease Initiation

LS is a multifactorial dermatosis arising from genetic predisposition, environmental factors, infections, hormonal changes, and immune system irregularities (Table 1). Other immune-mediated skin diseases, including psoriasis, pemphigus vulgaris, lichen planus, and hidradenitis suppurativa share these triggers. While overlapping nonspecific immune stimuli drive these conditions, they are distinguished by the specific immune pathways they activate, which lead to unique histological and clinical characteristics [18,28,32,33].

#### 3.1.1. Genetic Predisposition

Several studies document a genetic predisposition to LS, with approximately 12% of first-degree female relatives being affected [34]. Case reports of LS in monozygotic and dizygotic twins further support this genetic association [35,36,37]. However, in men, no clear genetic correlation has been found, suggesting that LS’s inheritance patterns may differ by gender [19]. Despite this, familial cases of LS are associated with an increased risk of vulvar carcinoma and other autoimmune disorders [16].

Human leukocyte antigen (HLA) class II genotypes are linked to both susceptibility and protection in LS. HLA-DQ7, for example, is frequently associated with LS in both sexes, with 50% of adult women and 66% of prepubertal girls with LS carrying this genotype, compared to 31% of controls [38,39]. While some HLA alleles, such as DR17, may confer protection, no correlation has been found between HLA type and disease onset, lesion location, or response to treatment [28,39].

Epigenetic changes also play a crucial role in LS pathogenesis. Early-stage LS shows minimal cell-cycle disruption, with no mutations in p53 or CDKN2A. Increased methylation of p16INKa gene promoters precedes p53 mutations, contributing to abnormal cell proliferation and tumour development, particularly vulvar carcinoma. Alterations in isocitrate dehydrogenase further reduce methylation levels in LS epidermis, and treatments like ultraviolet A1 (UVA1) have shown therapeutic potential by correcting these epigenetic changes [28,31,40].

#### 3.1.2. Environmental Trigger

##### Hormonal Factors

The role of hormones in LS remains debated. Although hypoestrogenism was once thought to increase LS risk, evidence supporting this is limited [28,41]. Studies show no significant differences in oestrogen receptor isoform expression between LS patients and healthy controls [28].

However, reduced androgen levels, potentially due to decreased 5-alpha-reductase activity or androgen receptor malfunction, have been linked to LS progression [16,28]. Evidence suggests that antiandrogenic oral contraceptives can disrupt hormonal balance and possibly increase susceptibility to LS. In contrast, pure progesterone preparations appear to have a protective effect, possibly by maintaining hormonal homeostasis and preserving the integrity of vulvar tissue [28,42,43,44]. Anti-androgenic oral contraceptives may also increase LS risk, whereas progesterone-only pills seem to have a protective effect [28,45]. Although topical progesterone can induce remission in premenopausal LS, ultrapotent corticosteroids remain the preferred treatment [28].

##### Immunological Abnormalities

Although the aetiology of LS is unknown, there are several indications that autoimmunity plays an important role in the pathogenesis of LS [3]. Autoimmune mechanisms are strongly implicated in the pathogenesis of LS, with studies reporting that between 21.5% and 34% of affected individuals also have at least one other autoimmune disease [32,46]. In a retrospective cohort study conducted in Germany involving 532 patients (396 women and 136 men; mean age 49 years) attending a specialised vulva clinic, 28.4% of women with LS had a co-existing autoimmune disease, compared to 8.8% of men. This gender disparity is supported by other studies showing that women with LS are more likely than men to develop an autoimmune disease [47]. Autoimmune thyroid disease is most commonly reported, affecting approximately 12.3% of patients, followed by alopecia areata (5.6%), localised scleroderma (4.2%), and psoriasis (3.8%) [47]. There is also a notable association in the paediatric population: in a cohort of 210 children with LS, 24.5% had autoimmune comorbidity, a figure that rose to 34.6% in an adult cohort of 381 patients. Autoimmune thyroiditis remained the most common diagnosis in both age groups [48]. Despite that, the current literature regarding the systematic screening of autoimmune comorbidities in the relevant patient group is still limited and unclear. Many recent studies have highlighted that, while certain autoimmune conditions have associations with the underlying condition, universal screening in the absence of clear risk stratification may lead to unnecessary testing and inefficient use of resources [28,48].

Another hallmark of LS is increased collagen synthesis in the dermis, particularly types I and III collagen. This sclerotic tissue formation is driven by the upregulation of miR-155, which downregulates tumour suppressor genes like FOXO3 and CDKN1B, leading to increased fibroblast proliferation. Additionally, anti-BP180 and anti-BP230 antibodies, commonly found in later stages of LS, are more prevalent in women, mirroring the higher incidence of autoimmune diseases in females [28,31]. These antibodies are associated with more severe vulvar LS lesions [49].

**Figure 2:**
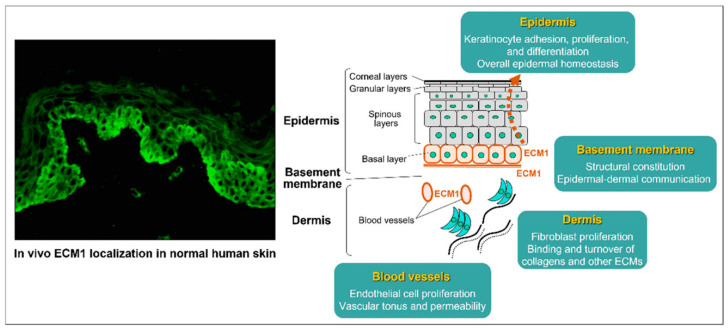
The diverse involvement of ECM1 in vivo with the surrounding extracellular matrix and structural elements of normal human skin, particularly in the epidermal basal layer, basement membranes, blood vessel walls, and follicular epithelium. The immunostaining on the left shows the presence of ECM1, while the right illustrates its crucial role in strengthening the integrity and maintaining the skin’s homeostasis. Reproduced from Oyama, N., Hasegawa, M.; “Lichen Sclerosus: A Current Landscape of Autoimmune and Genetic Interplay”. Diagnostics, MDPI, 2022, under CC BY 4.0 licence [50].

Autoantibodies targeting extracellular matrix protein 1 (ECM1) are another key feature of LS, particularly in males. ECM1 is a glycoprotein that maintains skin structure by binding various extracellular components (Figure 2). Autoantibodies targeting ECM1 impair its regulatory function, leading to increased collagenase activity and triggering a cascade of pathological changes. These include dyskeratosis and thinning of the epidermis, homogenisation of collagen fibres in the dermis, and the development of telangiectasias with increased vascular permeability. Overall, these changes contribute to the progressive damage to the skin and sclerosis of the dermis that characterises LS [28,31,50].

In cancer patients treated with immunotherapy and checkpoint inhibitors, there is a positive association with autoimmune-induced diseases, including LS. Some reports indicate that genital LS may occur about 3–5 months after starting treatment. However, the time of onset and frequency vary considerably between patient groups and treatment protocols. Given this variability, the current consensus emphasises the need for an individualised clinical assessment and management strategy [28].

##### Trauma and Chronic Irritation

Trauma and chronic irritation, such as occlusion, scratching, friction, or surgical procedures, can induce the Koebner phenomenon, where new LS lesions form at sites of skin injury. Oxidative stress is critical in LS pathogenesis by generating reactive oxygen species (ROS) that damage tissues, trigger autoimmunity, and promote scarring. ROS may also drive the production of autoantibodies against ECM1, perpetuating the cycle of inflammation and tissue damage [32]. Chronic exposure to urinary irritants may exacerbate LS [6], particularly in older women with urinary incontinence and in men with anatomical abnormalities or post-surgical complications. This emphasises the importance of managing underlying conditions that could contribute to skin irritation in LS patients [28].

In the context of LS, chronic inflammation produces ROS, resulting in tissue damage, oxidative stress, and various consequences such as lipid peroxidation, DNA and protein damage, tissue sclerosis, and scarring. ROS-induced skin epitopes can trigger autoimmunity, tumorigenesis, and dermal vasoconstriction. An important consequence of ROS is the development of new skin epitopes, which trigger the production of autoantibodies, mainly directed against ECM1 [18,28,31,32].

##### Infections

Although no specific infections are definitively linked to LS, certain pathogens such as human papillomavirus (HPV), hepatitis C (HCV), and *Borrelia burgdorferi* have been suggested as potential triggers [18,28,32]. Some reports have also speculated that SARS-CoV-2 could be a contributing factor, but this association remains unconfirmed [18]. LS patients are more susceptible to skin and gut microbiota shifts, possibly due to the underlying inflammatory processes, which could contribute to disease exacerbation [18].

### 3.2. Disease Maintenance

**Figure 3:**
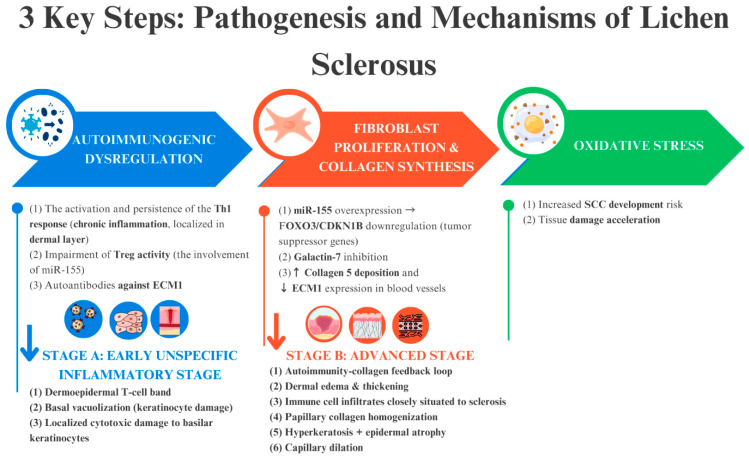
The trio of key pathogenic mechanisms underlying the onset and progression of LS—autoimmune dysregulation, sclerotic tissue formation, and oxidative stress—form an interconnected cycle that perpetuates the disease. The blue and red arrows indicate events that occur due to the above-mentioned factors.

The pathophysiology of LS remains poorly understood due to the involvement of numerous complex and interrelated processes. Research is ongoing to elucidate these mechanisms and to establish a clearer classification of LS subtypes and stages. The pathogenesis of LS can be summarised by three primary mechanisms: (i) inflammation and activation of signalling pathways that lead to autoimmunity; (ii) formation of sclerotic tissue; and (iii) oxidative stress (Figure 3). These processes collectively drive dermal fibrosis and the characteristic histopathological features of LS, which are critical for diagnosis [6,18,28,31,32].

#### 3.2.1. Autoimmunogenic Dysregulation

A breakdown in self-tolerance plays a critical role in developing LS, fostering an autoimmune environment [31,32]. A dermo–epidermal band of T-cells, basal vacuolization, and cytotoxic damage to basal keratinocytes characterise the initial nonspecific inflammatory phase. This autoimmune response is sustained through three primary mechanisms: (i) prolonged activation of the T helper 1 (Th1) cell response; (ii) involvement of microRNA-155 (miR-155); and (iii) dysfunction of ECM1 [6,18,28,31,32]. These processes together form the foundation of the cascade of immune reactions that drive LS pathogenesis.

T-cell infiltration, along with the resulting cytotoxic activity, plays a pivotal role in initiating, maintaining, and intensifying chronic inflammation [18]. This infiltration is mainly in the dermal layer and is mostly made up of CD8+ T cells and CD4+ regulatory T cells (Tregs), with a lesser number of conventional CD4+ T helper cells [28,32,41]. The chemokine receptor profile of these T cells notably includes CXCR3 and CCR5, with the absence of CCR3 and CCR4 suggesting a strong Th1-mediated response [18,28,31,32]. This immune response is characterised by the upregulation of proinflammatory cytokines, including IL-1α, IL-7, IL-15, and TNF-α, as well as immune mediators such as IL-2 receptor (CD25), caspase 1, ICAM-1, and its ligand CD11a [18]. Concurrently, there is a downregulation of anti-inflammatory cytokines like IL-10 [18,28,31]. The production of interferon-γ further enhances the recruitment of Th1 cells to the affected areas, exacerbating the inflammatory response [6,18,28,31,32].

The impairment of Treg activity, which is crucial for maintaining immune tolerance and preventing autoimmunity, is believed to be linked to dysregulated microRNAs, particularly miR-155. MicroRNAs are small endogenous RNA molecules that regulate gene expression by binding to the 3′ untranslated region (3′ UTR) of target mRNA transcripts. Dysregulation of microRNA expression is considered a significant factor in LS development. Specifically, miR-155 is overexpressed in activated immune cells such as macrophages, dendritic cells, B cells, and T cells. It plays a key role in controlling the production of cytokines, chemokines, and transcription factors, promoting Th1 differentiation and contributing to dermal sclerosis [28,31]. Blood samples from LS patients have shown significantly reduced levels of the transcription factor Foxp3, a marker for Treg cells, and decreased circulating levels of CD127, a phenotypic marker for CD4+ CD25+ Treg cells. However, despite these functional changes, there is no detectable reduction in the overall Treg cell count. This suggests that disruptions in Treg function—rather than their number—may trigger autoimmune reactions in LS [28,31,32].

**Figure 4:**
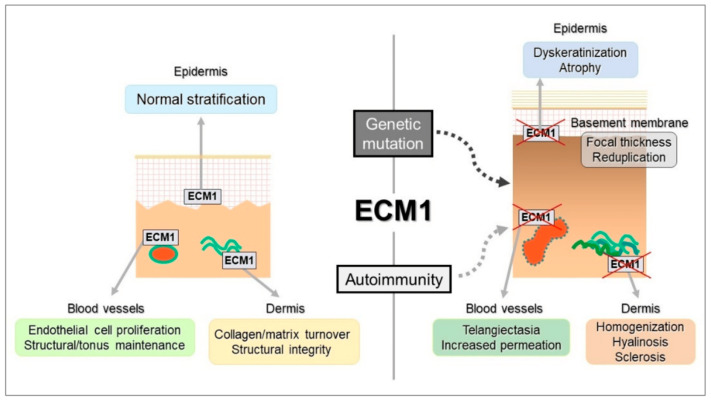
The schematic illustrates how autoimmune and genetic disruptions affect ECM1 function, leading to dysregulation of its binding partners. This contributes to a homeostatic imbalance in the epidermis (manifesting as dyskeratosis and atrophy), dermis (characterised by collagen homogenization and sclerosis), and blood vessels (resulting in telangiectasia and impermeability). Reproduced from Oyama, N., Hasegawa, M.; “Lichen Sclerosus: A Current Landscape of Autoimmune and Genetic Interplay”. Diagnostics, MDPI, 2022, under CC BY 4.0 licence [50].

Dysfunction in ECM1, triggered by autoantibodies, also contributes to disease progression [31,32]. When antibodies target ECM1-MMP9 binding, they induce excessive MMP9 activity, leading to the cleavage of TGF-β and subsequent activation of collagen synthesis. This imbalance in collagen production may explain the localised thickening of the basement membrane in certain regions of LS lesions, which is similar to mechanisms seen in lipid proteinosis (Figure 4) [28,31,32,50]. Although ECM1 autoantibodies affect different epitopes, their primary influence appears to be on the interaction between ECM1, collagen IV, and perlecan [31,32]. Autoantibodies against other basement membrane zone (BMZ) antigens, such as BP180 and BP230, are also common in advanced LS stages [18]. These antibodies are thought to play a role in disease progression rather than initiation—a phenomenon known as epitope spreading, where hidden antigens are exposed following tissue damage, triggering further autoimmune activity [18,28,31,32,50].

#### 3.2.2. Fibroblast Proliferation and Increased Collagen Synthesis Leading to Sclerotic Tissue Formation

One hallmark of LS is the increased proliferation of fibroblasts and the excessive synthesis of collagen, specifically collagen types I and III, in the dermis. This formation of sclerotic tissue is driven by three primary mechanisms: first, the upregulation of miR-155, which leads to the downregulation of tumour suppressor genes such as FOXO3 and CDKN1B, thereby promoting fibroblast proliferation; second, galectin-7, a pro-apoptotic keratinocyte protein, inhibits fibroblast growth while paradoxically increasing collagen synthesis; and third, within the blood vessels in LS-affected areas, there is increased deposition of collagen type V alongside a reduction in ECM1 expression, resulting in a decreased content of elastic fibres [18,28,31,32].

The interplay between dysregulated autoimmunity and abnormal collagen metabolism is a key catalyst that triggers their mutual activation, creating a feedback loop that amplifies the pathological effects. Histopathological studies of LS lesions confirm this dynamic relationship, showing immune cells infiltrate close to sclerotic areas [18,31]. Chronic inflammation persists, leading to dermal oedema and the uniform arrangement of papillary dermal collagen fibres, eventually resulting in hyperkeratosis [18,32]. In less common cases, epidermal atrophy may occur with the flattening of the rete ridges, accompanied by dilation of the papillary capillaries.

The overexpression of miR-155 not only downregulates critical tumour suppressor genes, such as FOXO3 and CDKN1B, but also plays a well-documented pro-fibrotic role in various fibrotic conditions by promoting the synthesis of ECM proteins, including collagen [18,28,31,32]. Although the precise mechanisms by which miR-155 drives fibroblast proliferation and fibrosis in LS are still unclear, it is likely that miR-155 affects multiple signalling pathways and gene expressions, thereby triggering abnormal fibroblast proliferation and increased cell cycle activity, ultimately leading to persistent tissue scarring [18,28,31,32].

Additionally, fibroblast activity is further stimulated by elevated levels of galectin-7, which is regulated by p53 [18,29,31]. Galectin-7 exerts its effects through two primary mechanisms: first, by downregulating the growth rate of epidermal cells; and second, by inhibiting the viability of dermal fibroblasts while promoting the transcription of type I and type III collagen [18,28,31,32]. The activation of galectin-7 triggers the release of cytochrome c and activates JUN N-terminal kinase (JNK), leading to programmed cell death and further inhibiting cellular viability. Galectin-7’s potential to enhance apoptosis in epidermal cells contributes to reduced keratinocyte viability, resulting in epidermal atrophy, contraction, and depletion of epidermal tissue [18,28,31,32,51].

It has been hypothesised that the increased expression of galectin-7 may be secreted into the dermal layer, where it potentially interacts with fibroblasts, influencing their function and resulting in the upregulation of collagen types I and III [31,51]. Further research is required to determine whether galectin-7 plays a role in the irregular distribution of hyalinized collagen fibres, as observed in LS. Additionally, galectin-7 may promote T-cell viability, further contributing to the pathological processes in LS [18,28,31,32,51,52].

#### 3.2.3. Oxidative Stress

Low levels of antioxidant enzymes, such as superoxide dismutase, have been observed in keratinocytes of chronic LS lesions. This deficiency is closely linked to increased lipid peroxidation, oxidative DNA damage, and protein oxidation. These processes contribute to cellular dysfunction and tissue damage, exacerbating the disease’s progression [17,28,31,32,51].

Oxidative DNA damage further contributes to epigenetic changes in the CDKN2A gene, leading to the downregulation of two key cyclin-dependent kinase tumour suppressors, p16INK4 and p27Kip1 [17,31]. This cascade of molecular events may facilitate the development of squamous cell carcinoma in LS patients [17,18,28,31,32].

Oxidative stress, primarily driven by the generation of ROS, creates new epitopes that can trigger autoimmune responses [31,32]. This stress also promotes the overexpression of wild type p53 in basal keratinocytes, a compensatory mechanism aimed at counteracting the damaging effects of oxidative stress [28,31,32]. Additionally, the development of sclerotic blood vessels and the resulting hypoxic environment further exacerbate oxidative stress, maintaining a cycle of ischemic stress that perpetuates local oxidative damage. This environment enhances p53 expression, leading to increased protein stability and accumulation, potentially serving as a defence mechanism against oxidative injury [17,18,28,31,32].

Although mutations in p53 and CDKN2A were not detected, the focus has shifted toward understanding the role of epigenetic modifications in LS. These alterations are crucial in triggering malignant transformation, highlighting the importance of epigenetic regulation in disease progression and potential cancer development in LS patients [28].

## 4. Challenges in Diagnosis

Diagnosis of LS is primarily based on clinical assessment, including medical history and physical examination. Screening for autoimmune comorbidities, particularly thyroid disease, is recommended if clinical signs or symptoms are apparent. Histopathological examination is typically reserved for cases where malignancy is suspected or when distinguishing LS from similar conditions like vulvitis, eczema, or lichen planus [18,28].

Although clinical examination and histopathological assessment remain the cornerstones of LS diagnosis, the potential for malignant transformation by precursor lesions, such as differentiated vulvar intraepithelial neoplasia (dVIN), is a major concern, particularly in women. dVIN is a non-HPV-related high-grade dysplasia that often occurs in association with chronic inflammatory dermatoses, particularly LS, and is considered a high-risk precursor to vulvar squamous cell carcinoma (VSCC). The estimated absolute risk of VSCC after solitary dVIN treatment is approximately 33–43%, with a median time to development of invasive cancer of 25.4 months. The diagnosis of dVIN is difficult due to the histological overlap with benign vulvar diseases such as psoriasis, lichen planus, and LS itself. In addition, corticosteroid therapy can obscure important diagnostic features, making early detection difficult [53]. Although histopathology remains the diagnostic gold standard, biopsy is recommended not only for atypical findings or diagnostic uncertainty, but also proactively to identify dVIN, to allow timely surveillance and therapeutic intervention. Early detection and treatment with highly effective topical corticosteroids can reduce the risk of malignant progression in LS-associated dVIN [32,54].

**Figure 5:**
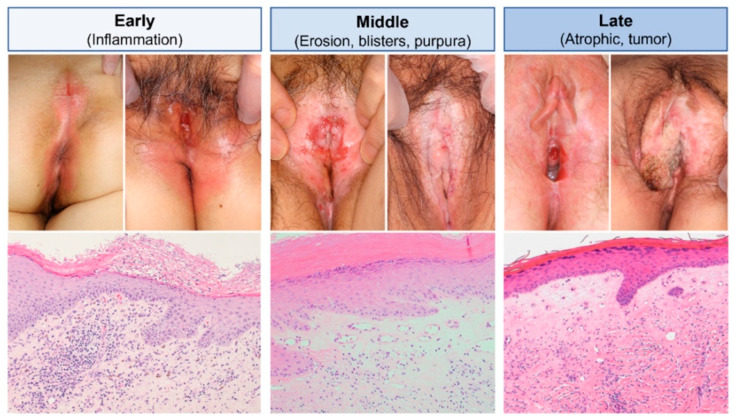
Stage-specific clinical and pathological manifestations. In the initial clinical phase (**left column**), LS presents with erythema. Histopathologically, early LS is characterised by parakeratotic scales, irregular epidermal thickening, subepithelial inflammatory infiltrates, and homogenization of dermal collagen bundles. In the intermediate phase (**middle column**), persistent erosions with focal blistering and purpura develop, accompanied by whitish-pale indurative polygonal papules and plaques. Dilated blood vessels can be seen just below the basement membrane. In advanced clinical stages (**right column**), the vessels narrow and eventually disappear as dermal fibrosis outpaces vascular changes, leading to scarring and irreversible adhesion of external genital components. In extreme cases, this can result in the sudden onset of squamous cell carcinoma. Reproduced from Oyama, N., Hasegawa, M.; “Lichen Sclerosus: A Current Landscape of Autoimmune and Genetic Interplay”. Diagnostics, MDPI, 2022, under CC BY 4.0 licence [50].

Although histopathological examination has certain limitations, it remains the gold standard for diagnosing LS and reveals distinctive features that vary with the disease stage (Figure 5). The hallmark histological findings encompass changes in the epidermis, dermis, and adnexal structures [18,28].

The epidermis is often thinned and exhibits atrophic features, particularly in advanced stages [32,50]. Orthokeratosis, frequently accompanied by follicular plugging, is a common observation [28,32,55]. Basal keratinocyte degeneration is a critical early marker, signifying disruption at the dermo–epidermal junction. Dermal changes in LS are prominently observed in the papillary dermis, where a band-like inflammatory infiltrate, predominantly composed of lymphocytes, is a key feature in early stages [18,28,31]. As the disease progresses, the inflammation often extends into the deeper dermis [28]. A distinctive hallmark in the mid-dermis is the homogenization or sclerosis of collagen fibres, producing a hyalinized, glassy appearance. This feature is diagnostically significant and aids in distinguishing LS from other similar dermatoses [31,56]. In chronic cases, the appendages disappear [28]. The vascular changes are equally significant. Capillaries and small blood vessels commonly exhibit dilation in active lesions, reflecting ongoing inflammatory processes. In late or resolved lesions, normal vessels are notably absent [28].

To ensure accurate diagnosis and monitor disease progression effectively, it is recommended to perform multiple biopsies from active sclerotic areas and non-healing erosions, particularly in cases where lesions are resistant to treatment. Regular follow-ups and biopsies can help identify changes and adjust treatment plans as necessary [28,31].

Photographic documentation can be highly useful for tracking the effectiveness of therapy and the progression of the disease. Dermoscopy has become a valuable, non-invasive diagnostic tool to optimise biopsy site selection and improve diagnostic accuracy. In the early stages of LS, dermoscopy typically reveals irregular linear vessels, observed in approximately 97% of cases, alongside dotted vessels in about 45% of cases. As the disease progresses, dermal fibrosis leads to the gradual disappearance of these vascular patterns. Additional features include scattered grey-blue dots, comedo-like openings, and fine scales. White, patchy lesions on an atrophic background are also characteristic, reflecting the chronic nature [28,57,58,59].

**Figure 6:**
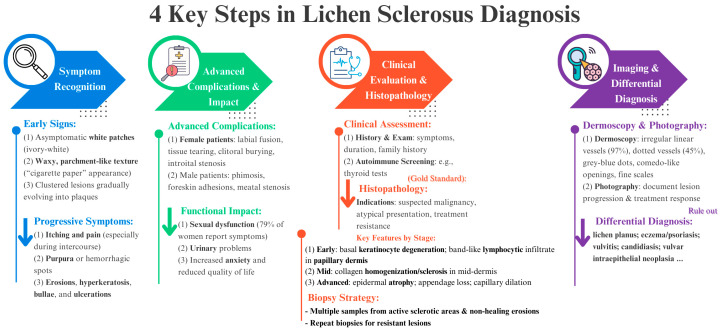
Systematic diagnostic approach to LS, emphasising the four key steps from symptom awareness through clinical examination, imaging studies, and differential diagnosis, illustrating the multifaceted process necessary for precise diagnosis and best patient management. Each color arrow represents key points that can assist clinicians at each step of LS diagnosis.

Figure 6 provides a practical framework to assist clinicians in the diagnostic assessment of LS. It outlines a stepwise approach that includes identification of symptoms (from early signs to advanced complications), clinical examination and histopathological confirmation (with biopsy as the diagnostic gold standard), imaging and differential diagnosis (including dermoscopy and clinical photography), and consideration of the impact of the disease on the patient’s quality of life. This flowchart is designed to facilitate a rationalised and comprehensive diagnostic process.

Recent reviews emphasise the importance of a comprehensive differential diagnosis in vulvar lichen sclerosus (VLS), as there are clinical overlaps with diseases such as lichen planus, chronic candidiasis, vitiligo, and vulvar intraepithelial neoplasia (VIN). Accurate differentiation requires a multimodal approach that combines a detailed clinical examination, targeted biopsy of atypical or treatment-resistant lesions, and complementary imaging techniques such as dermoscopy and immunofluorescence tests. This methodical exclusion of mimicking conditions is essential to ensure timely and accurate diagnosis, appropriate therapeutic intervention, and a reduction in diagnostic delays [28,60].

Ultrapotent topical corticosteroids, in particular clobetasol propionate 0.05%, remain the cornerstone of VLS treatment. In addition to symptom relief, clobetasol has anti-inflammatory and anti-fibrotic effects by modulating Th1-mediated immune responses and inhibiting dermal collagen deposition, thereby mitigating scarring and potentially reducing the risk of malignant transformation. There is evidence that long-term, structured use of clobetasol may reduce the incidence of vulvar squamous cell carcinoma (VSCC), particularly in patients who adhere consistently to treatment. Given the chronic and potentially progressive nature of VLS, regular follow-up is essential. Current guidelines recommend clinical examinations every three to six months for the first two years after diagnosis, and annual examinations thereafter. This surveillance strategy facilitates the monitoring of treatment response, adjustment of maintenance therapy, early detection of recurrences, and identification of pre-invasive lesions such as differentiated VIN (dVIN), thus optimising long-term outcomes [28,60].

## 5. Conclusions

Genital LS is a common condition, making early detection and prompt treatment critical. The disease has a significant impact on quality of life, impairing sexual activity and carrying the potential for malignant transformation. LS primarily affects the genital, perineal, and perianal areas and presents as patchy, thin, and ivory-white lesions. With an estimated prevalence of 1 in 300 to 1 in 1000 people, LS is on the borderline between common and rare diseases. This epidemiological ambiguity underlines the importance of increased clinical awareness and diagnostic vigilance to ensure timely detection and appropriate treatment.

Although the exact pathogenesis of LS remains unclear, it is understood to involve genetic predisposition, immune dysregulation, fibroblast proliferation, collagen synthesis, and oxidative stress. Diagnosis typically relies on clinical assessment, with biopsies reserved for specific cases where differentiation is necessary.

Continued research is essential to further unravel the complexities of LS and to develop targeted therapeutic strategies. Advances in molecular techniques, particularly those aimed at modulating specific pathogenic pathways, offer hope for altering the disease’s course, similar to the progress made in treating psoriasis and atopic dermatitis.

## Figures and Tables

**Table 1:** Initiating factors for LS.

Trigger	Description
**Genetic predisposition**	− Higher LS incidence in female twins and family members − Strong association with HLA-DQ7
**Epigenetic alterations**	− Minimal cell-cycle disruption without p53/CDKN2A mutations − Increased p16INKa methylation precedes p53 mutations, contributing to abnormal cell growth and carcinoma risk
**Hormonal changes**	− Low androgen levels linked to LS progression − Anti-androgenic contraceptive pills may increase risk, while progesterone-only pills may be protective
**Immunological abnormalities**	− Linked to autoimmune diseases (e.g., thyroid disorders) − Autoantibodies (anti-BP180 and anti-BP230) present in later stages of LS
**Local factors**	− Trauma triggering the Koebner effect − Urine as an irritant in urogenital areas − Oxidative stress leading to autoantibody production, primarily targeting ECM1
**Infections**	− Possible involvement of HPV, HCV, and Borrelia burgdorferi − SARS-CoV-2 link has been suggested but not proven

## Data Availability

The original contributions presented in this study are included in the article. Further inquiries can be directed to the corresponding author.
